# Differences in center of pressure trajectory between normal and steppage gait

**Published:** 2010

**Authors:** Nima Jamshidi, Mostafa Rostami, Siamak Najarian, Mohammad Bagher Menhaj, Mohammad Saadatnia, Firooz Salami

**Affiliations:** a Research Associate, Department of Biomechanics, Faculty of Biomedical Engineering, Amirkabir University of Technology, Tehran, Iran; b Associate Professor, Department of Biomechanics, Faculty of Biomedical Engineering, Amirkabir University of Technology, Tehran, Iran; c Professor, Department of Biomechanics, Faculty of Biomedical Engineering, Amirkabir University of Technology, Tehran, Iran; d Professor, Faculty of Electrical Engineering, Amirkabir University of Technology, Tehran, Iran; e Associate Professor, Department of Neurology, Isfahan University of Medical Sciences, Isfahan Medical Education Research Center, Isfahan Neuroscience Research Center, Applied Physiology Research Center, Isfahan, Iran

**Keywords:** Classifications, Steppage Gait, Neuromuscular Disease, Force Plate

## Abstract

**BACKGROUND:**

This pilot study aimed to assess the differences in center of pressure trajectory in neuropathic patients with steppage gait. Steppage gait has previously been evaluated by several biomechanical methods, but plantar pressure distribution has been much less studied. The purpose of this study was to analyze the changes in center of pressure trajectory using a force plate.

**METHODS:**

The steppage gait group was selected from the patients using drop foot brace (25 male) and the control group was selected from Isfahan university students (20 male). They walked at self- selected speed at a mean of ten trials (+2) to collect the center of pressure using a force plate. Center of pressure patterns were categorized into four patterns based on the center of pressure displacement magnitude (spatial features) through time (temporal features) when the longitudinal axis of the insole was plotted as the Y- axis and the transverse axis of the insole as X- axis during stance phase.

**RESULTS:**

The horizontal angle measured from center of pressure linear regression was positive in the control group (4.6 ± 2.4) (p < 0.005), but negative in the patient group (- 2.3 ± 1.6) (p < 0.005).

**CONCLUSIONS:**

The finding of this research measured center of pressure trajectory in steppage gait over time, which is useful for designing better shoe sole and also orthopaedic device and better understanding of stability in patients with drop foot.

The dynamic study of bipedal locomotion of normal and pathological human subject is enhanced by an analysis of joints kinetics data including muscle force and joint reaction force. During major part of normal walking, body weight is supported by one limb (stance phase) and this part of gait demonstrates several capabilities such as muscular coordination, balance, strength and joint kinematics.^1^ In fact extrinsic measures of musculoskeletal loading play a major role in the identification of injury mechanisms during gait. Direct measurement of the ground reaction force using a force plate is a common practice for the calculation of intersegmental forces and moments.^2^

Ground reaction force data obtained from a force platform or force plate, which is a transducer set into the floor to measure the forces and torques applied by the foot to the ground.^3^ Measuring the ground reaction forces between body and the supporting surface could be included valuable information.^4^ Center of pressure (COP) is the point of location of the vertical ground reaction force vector.^5^ Understanding center of pressure spatial relationship relative to the location of primary joints in normal gait is intuitively helpful in understanding the pathomechanics of a given patient. When both feet are in contact with the ground, the location of COP under each foot reflects the neural control of the ankle muscles. COP moves to the anterior with the increased activity of the plantar flexors and it moves laterally with the increase in invertor muscles activity.^5^ In previous research COP has been used as a good index to calculate the balance of individuals.^6^–^9^ COP would be useful in motion evaluation and clinical application.^10^–^14^ Another study revealed that the COP relations to foot pathology could be used to torques calculation about the joint axis of the foot.^15^ Previous studies have also indicated the use of COP to estimate an index to evaluate the function of rehabilitation devices such as foot orthoses during walking.^16^–^19^

Abnormal gait may be due to an injury, disease, pain or problems of motor control.^20^ Drop foot is an abnormal neuromuscular disorder characterized by steppage gait.^21^ People with drop foot always have difficulty in walking. Center of pressure can be used as a measure of dynamic postural control in a variety of normal and neuropathic subjects. A better understanding of center of pressure movement (pressure distribution) during walking will facilitate clinicians’ assessment and enhance treatment in patients with drop foot and can provide information about postural control in both normal and pathological situations. Since steppage gait affects the physical movement of lower extremity, this study primarily aimed to examine the changes in COP pattern. In addition, the study estimated the differences between normal and abnormal subjects during walking. A more detailed description of steppage gait can be derived from COP time series measurements taken at each foot during the gait cycle. These measures may reflect aspects of the underlying motor control for walking and have been used to characterize differences in steppage gait. The goal of this study was to identify differences of COP patterns in normal and steppage gait. However, relatively few studies have looked at COP patterns during steppage gait. There has been limited research utilizing this technique under dynamic conditions in those with drop foot. The clinical application of force plate data especially center of pressure analysis in steppage gait is novel. The finding of this research may help us to expand the use of force plate data to quantify steppage gait over time. One of the purposes of this observational study is to determine whether it is possible to detect changes in the gait cycle in patients with steppage gait using center of pressure pattern.

The numerical analysis of such data is useful for designing better shoe sole and also orthopaedic device especially for drop foot patients. Understanding the differences in center of pressure trajectory may lead us to have better understanding of stability in patients with drop foot and finally more efficient shoe sole design for them. COP trajectory times series data would help us to find the exact place of exerting the kinetics data to three dimensional finite element model of lower extremity during stance phase. By changing the thickness and materials of different layer of sole in rehabilitation devices the tension variations has been dynamically and continuously assessed. The tension reduction in sole can improve the effect of rehabilitation devices during abnormal gait.

## Methods

**Figure 1:**
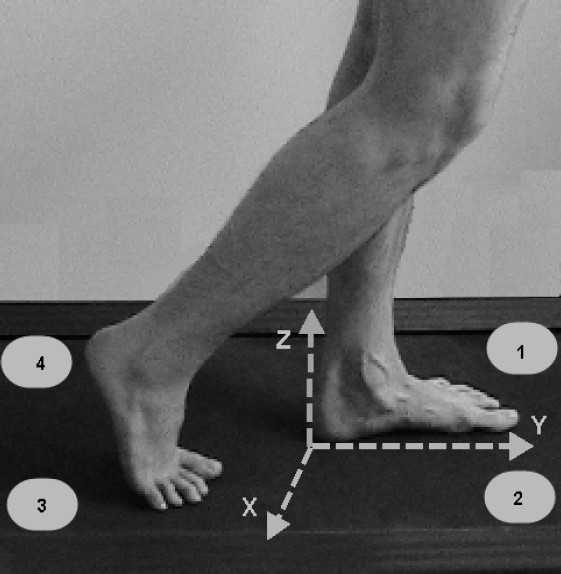
Schematic of Kistler coordinates system.^24^ (with kind permission from publisher)

The present study examined the time- domain parameters of ground reaction force components using a straingauge force platform sized 400×600 mm (model 9286AA, Kistler Group, Winterthur, Switzerland) based on piezoelectric sensors. Data were sampled at the rate of 1 kHz and an appropriate associated software was used for data analysis.^22^ As shown in figure 1, the vertical force in the Kistler coordinate system is calculated by equation 1 if walking direction is positive Y-axis; fzi is the measured force in z direction by sensor i (i = 1…4).^23^ (1) FZ = fz1 + fz2 + fz3 + fz4

**Figure 2:**
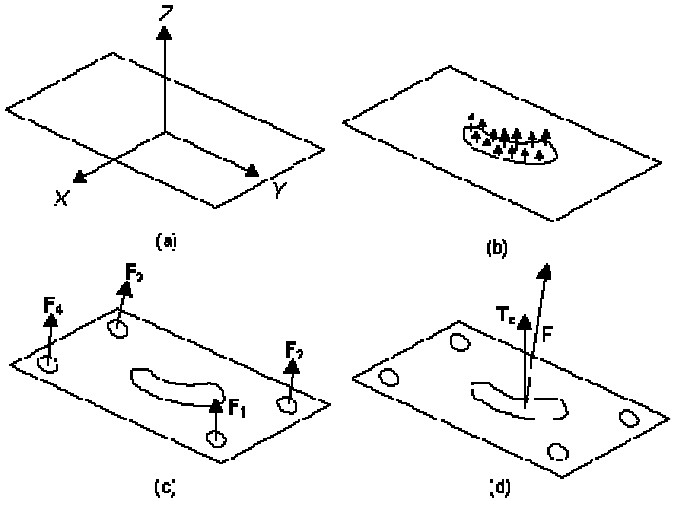
Schematic of coordinate and force plate calculation of vertical force and COP: a) Kistler coordinate system, b) pressure distribution of foot, c) forces captured from force plate sensors and d) final force and torques captured from force plate.^26^ (with kind permission from young-Hoo Kwon)

Following force variables were measured from the vertical components of the ground reaction force data. The base of the ground reaction force vector lies within the foot, as that is the body segment in contact with the floor. This point is called the center of pressure. By tracking the path of the instantaneous center of pressure during stance, the patient’s balance and pattern of progression can be determined. Each center of pressure point represents the mean of the vertical forces on four points.^25^ As shown in figure 2, all the forces acting between the foot and the ground can be summed to yield a single ground reaction force vector (*F*) and a free torque vector (*Tz*). The point of application of the ground reaction force on the plate is the COP.^26^ By tracking the path of the center of pressure during the stance phase, the pattern of progression can be revealed. In this study, force plate system was used to recognize differences in center of pressure between normal and neuropathic subjects.

Each participant was then given time to become familiar with the lab environment and was allowed a number of walking trials prior to data collection. Subjects performed ten trials for each foot at the participant’s normal walking speed. A valid trial consisted of the participant striking their heel on the force platform without altering their normal gait. Twenty normal and twenty five neuropathic subjects took part in the study. A subset of the database was used which contained 240 gait cycles of 20 normal male subjects (mean ± SD; age: 27.55 ± 10.6 years, mass: 67.72 ± 13.19 kg, height: 173.5 ± 5.89 cm) and 300 gait cycles of 25 male patients (mean ± SD; age: 30.24 ± 11.87 years, mass: 62.28 ± 14.42 kg, height: 168.48 ± 15.84 cm) with various gait problems due to Nerve injury - Sciatic Palsy (n = 11), Spinal Muscular Atrophy (n = 6), Spinal Tumor- Radiculopathy (n = 2), Nerve injury - Radiculopathy (n = 2), Guillain Barre syndrome (n = 1) and Charcot- Marie- Tooth disease type 2 double drop foot- inheretive- family disorder (n = 3). There were no differences in the age, weight, height and body mass index between the patients and the controls (p > 0.05). During normal walking, at the beginning of single stance phase, the center of pressure lies on the medial- posterior heel. Then it moves through the mid- foot region and continues towards the forefoot, crossing the metatarsal heads to terminate in the region of the great and the second toe. Significant distortions of this pattern can give evidence of abnormal loads on the foot and problems in the correct progression of the gait.^27^,^28^

**Figure 3:**
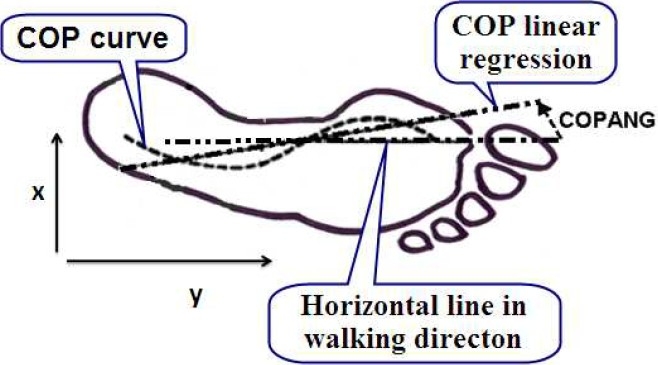
The horizontal X and vertical Y displacement of the instantaneous center of pressure (COP) during stance in the bottom of foot.

The (anterior- posterior) X and (medio- lateral) Y displacement of the instantaneous center of pressure during stance in the bottom of the foot has been shown in figure 3. CO- PANG represents horizontal angle measured from center of pressure linear regression and OPDEV represents center of pressure standard deviation from linear regression. The SPSS version 17 was used to analyze the statistical ground reaction force data. The data obtained from force plate were defined as means ± standard deviation. Two- tailed Student’s t test was run to clarify the differences between study and control groups. Differences with a significance level (p) lower than 0.05 were determined significant.

## Results

The COPANG and COPDEV are indices that show us how the statistical analysis is valid between normal and steppage gait (Table 1).

**Table 1:** List of vertical parameters derived from force plate measurements*

	Control Group (n = 20)	Patients (n = 25)	P Value
COPANG	4.581267 ± 2.444394	-2.31327 ± 1.621338	p < 0.005
COPDEV	0.7343 ± 0.28533	0.6061 ± 0.30675	p < 0.005

* Mean ± SD (standard deviation).

**Figure 4:**
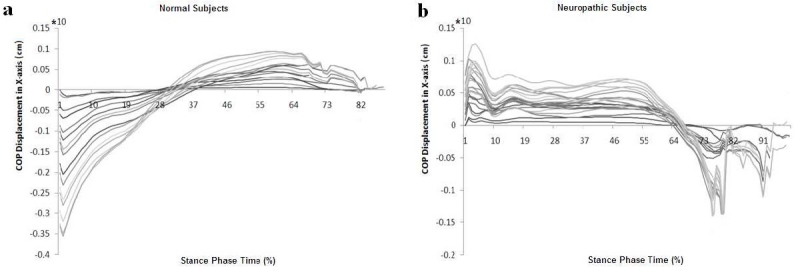
Diagram shows center of pressure patterns during a normal stride in X-axis direction during a normal stride among (a) normal and (b) neuropathic subjects with drop foot

The center of pressure patterns during a normal stride in X- axis direction shows a difference between normal and neuropathic subjects. As illustrated in figure 4, the slope of line between extreme of patients’ diagram is negative but it is positive in normal subjects.

**Figure 5:**
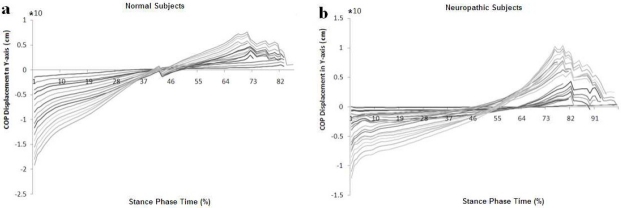
Diagram shows center of pressure patterns during a normal stride in Y-axis direction during a normal stride (a) normal and (b) neuropathic subjects with drop foot.

Also as shown in figure 5, the pattern of center of pressure trajectory among normal subjects in Y direction is similar to neuropathic subjects. This research revealed that the center of pressure patterns during a normal stride in Y- axis direction is almost the same in normal and neuropathic subjects, but the center of pressure patterns during a normal stride in X- axis direction is quite different.

## Discussion

This observational study has recorded the COP displacement in steppage gait. In this research, the center of pressure trajectory from force plate’s data was investigated. The focus of this research was on time series of data of COP trajectory. The present research clarified the differences in COP trajectory between normal and steppage gait. Table 1 provides information which reveals that the COP trajectories in the medio- lateral and anterio- posterior direction are reliable, because the COPANG and COP- DEV index in the medical research relies on p value and the p value in this research is less than 0.005. Results indicated a wide variation in the displacement of COP in the antero- posterior direction but not in the medio- lateral direction. The result of this research revealed that the pattern of center of pressure trajectory in anterior- posterior axis direction in neuropathic subjects is different compared to normal subjects. The extension and variation of ground reaction force database may complement the findings. Therefore, the difference in anterior- posterior COP trajectory patterns may result from pathologies in neuro muscular systems. Excessive different in anterior- posterior COP trajectory between normal and drop foot subject during gait may lead to a loss of balance. Results of a bipedal walking model study^29^ indicated that an active control from the nervous system is necessary to maintain frontal plane gait stability.^30^ Consequently, coordination of normal gait is supplied by neuro- musculoskeletal system, which relies on proper work nervous system, brain, spinal cord, nerves, as well as muscles, bones, cartilage, and joints of the body. Steppage gait is a form of gait abnormality which could be caused by damage to the peroneal nerve. This degraded nerve system feedback may affect their ability to control center of mass motion and result in a different antero- posterior COP pattern. Quantifying dynamic stability or postural perturbation requires an understanding of how the COP motion is generated and controlled continuously during locomotion.^31^,^32^ Deviations of an individual trace from the normative curve might depend on the intensity of gait disorder.

Force platform analysis of quiet standing offers a non- invasive, low- impact option to investigate postural control.^33^ Direct measurement of the ground reaction force using a force plate is common practice for the calculation of intersegmental forces and moments. While this type of measurement is certainly very accurate, it is difficult to implement in a natural environment outside motion analysis laboratory.^2^

Bobbert and Schamhardt34 evaluated the accuracy of determining center of pressure by applying forces through a known point of application to a Kistler force platform. Errors varied in magnitude up to ± 20 mm, depending on the position across the plate. Probably in the present research the effect of force plate error is very low because the normal of ground reaction force has been calculated accurately by using biomechanical model of subjects which has demonstrated by Bobbert et al^35^ in bio-ware software. COP trajectory patterns may be correlated with walking speeds and intensity of disorder because in some researches it takes into account. In the present research the cause of measurement errors such as inappropriate data smoothing^36^ has been omitted by using very popular hardware and software. Gait velocity is an effective index of the kinematic and kinetic measurements of human walking.^37^,^38^ In the present study the significant differences in COP trajectory pattern confirm that the gait velocity effect is ignorable.

## Conclusions

The finding of this research is useful for designing better shoe sole and orthopaedic device such as ankle foot orthosis through finite element analysis which exactly represents the place of exerting force. The center of pressure trajectory is useful for precise dynamic analysis of human gait and better understanding of stability in patients with drop foot. The required kinetics data for the three dimensional finite element model of lower extremity would be captured through force plate data such as magnitude and COP trajectory. By changing the thickness and materials of different layer of sole in ankle- foot- orthosis, the tension variations has been assessed. The effect of orthosis on tension generated in bones and muscles has been modelled. The tension reduction in sole can improve the effect of ankle- foot- orthosis during abnormal gait. It is possible to design each orthosis sole based on the kinetics data of each patient. However, due to small patient numbers and study being observational in nature, a larger database is needed. However, due to wide differences in COP trajectory pattern, this study has established clear relationship between drop foot and COP trajectory curve. Results demonstrated in this study could be an indicator of steppage gait. Taking these differences between normal and abnormal gait into consideration, this method of kinetic assessment, as indicated earlier, could be extended to detect the severity of the curve and gait dysfunction in drop foot subjects. Changing the antero- posterior COP trajectory may decrease the stability of neuropathic patients during the neuropathic gait.
